# Protective Effects of Fufang Xueshuantong on Diabetic Retinopathy in Rats

**DOI:** 10.1155/2013/408268

**Published:** 2013-09-24

**Authors:** Huihui Duan, Jianmei Huang, Wei Li, Minke Tang

**Affiliations:** School of Chinese Materia Medica, Beijing University of Chinese Medicine, Beijing 100102, China

## Abstract

The aim of this study was to evaluate the protective effects of Fufang Xueshuantong (FXT) on diabetic retinopathy in rats induced by streptozotocin (STZ). Diabetes was induced in Sprague-Dawley rats by a single injection of 60 mg/kg STZ. One week after STZ, FXT 0.525 g/kg or 1.05 g/kg was administrated to the rats by intragastric gavage (ig) once daily consecutively for 24 weeks. The control rats and untreated STZ rats received vehicle the same way. At the end of the experiment, the erythrocyte aggregation and blood viscosity were assayed. The retina vessel morphology was observed in retinal digestive preparations. Expression of occludin and intercellular adhesion molecule-1 (ICAM-1) in retina was measured by western blotting. Expression of vascular endothelial growth factor (VEGF) and pigment epithelium derived factor (PEDF) in retina was detected by immunohistochemistry. The activity of aldose reductase in retina was investigated with a NADPH oxidation method. The results showed that, in STZ rats, there were distinct lesions in retinal vessel, including decrease of pericytes and increase of acellular capillaries, together with dilatation of retinal veins. The expression of VEGF and ICAM-1 increased, while the expression of PEDF and occludin decreased. The activity of aldose reductase elevated, and the whole blood viscosity, plasma viscosity, and erythrocyte aggregation also increased after STZ stimulation. FXT 0.525 g/kg and 1.05 g/kg demonstrated significant protective effects against STZ induced microvessel lesion in the retina with increased pericytes and reduced acellular capillaries. FXT also reduced the expression of VEGF and ICAM-1 and enhanced the expression of PEDF and occludin in STZ insulted rats. The activity of aldose reductase, the whole blood viscosity, plasma viscosity, and erythrocyte aggregation also decreased after FXT treatment. The results demonstrated that FXT has protective effect on STZ induced diabetic retinopathy in rats.

## 1. Introduction 

Diabetic retinopathy (DR) is a leading cause of adult vision loss in developed countries and the prevalence of retinopathy is directly related to the duration of diabetes [1, 2]. The prevalence of DR is 50% and 90% in 10 years and 20 years diabetic patients, respectively [3]. The blindness triggered by diabetic retinopathy is 25 times more than that in non-diabetic retinopathy [4]. Therefore, it is highly needed to prevent and control diabetic retinopathy. As microvascular disease, diabetic retinopathy is characterized by pericytes loss, endothelial cells proliferating, capillary basement membrane thickening, increased microvascular permeability, edema, retinal vein dilation, microaneurysms, hemorrhages, and neovascularization [5–7]. DR is a dynamic process from non-proliferative diabetic retinopathy (NPDR) to proliferative diabetic retinopathy (PDR). Currently, NPDR is the focus of study and is also the best phase for drug intervention, because when it comes into PDR, the probability of blindness increases significantly and the possibility of recovery reduces greatly [8–10]. 

The blood-retinal barrier (BRB) plays a critical role in partitioning the neural elements of the retina from the circulation and protecting them from circulating inflammatory cells and their cytotoxic products [11]. Long-term and persistent hyperglycemia could damage the structure and function of retinal vessels through many pathways, which include the activated polyol pathway, increased formation of advanced glycation endproducts, enhanced formation of diacylglycerol, and activation of specific protein. The activation of polyol pathway is one of the key factors inducing pericytes to selective loss [12, 13], in which VEGF plays a vital role. Accumulating pieces of evidence suggest that overexpression of VEGF could not only damage the structure of BRB but also promote retinal angiogenesis. It has been reported that ICAM-1, which could be induced by VEGF, is a crucial mediator of vascular permeability [14, 15]. In addition, VEGF could damage the BRB by inducing rapid phosphorylation of occludin, which is an important tight junction protein to ensure the structural and functional integrity of BRB [1]. Normally, PEDF could inhibit the angiogenesis induced by VEGF in diabetic retinopathy. However, many researches have detected increased VEGF and decreased expression of PEDF in retina in diabetic rats, which suggested that the balance between VEGF and PEDF was disrupted [16]. STZ (single dose of 60 mg/kg) induced diabetic rats are commonly used as DR animal model. Many researches used STZ rats to study DR and other diabetic complications. Recently, it has been demonstrated that fortified extract of red berry, Ginkgo biloba, and white willoe bark, containing *α*-lipoic acid and L-carnosine, significantly reduced retinal levels of TNF-*α*, VEGF, and plasma TBARS in diabetic rats [11]. All these studies provide very promising therapeutic applications. Using the STZ model, in this study, we investigated the effect of Fufang Xueshuantong (FXT).

FXT is a widely used Chinese herbal formula in ophthalmologic clinical application, which consists of *Panax notoginseng*, *Salvia miltiorrhiza*, *Astragalus membranaceus*, and *Scrophularia ningpoensis*. It was found that FXT could dilate the blood vessels and promote blood circulation [17, 18]. A large number of clinical studies indicate that FXT could prevent fundus hemorrhage and microaneurysms in DR patients. However, its mechanism has yet to be determined. The aim of the study was to investigate the effects of FXT on the early stages of diabetic retinopathy induced by injection of STZ in rats.

## 2. Materials and Methods

### 2.1. Animals and Reagents

Male Sprague-Dawley (SD) rats were purchased from Vital River (China) at a weight of 230 to 250 g (SCXK (Jing) 2007-0001). Experiments performed in this study adhered to the Association for Research in Vision and Ophthalmology (ARVO) statement for the “Use of Animals in Ophthalmic and Vision Research.” Animal experimental protocol was approved by the BUCM Institutional Animal Care and Use Committee. Rats were acclimated for one week with a 12-hour light/dark cycle, constant temperature and humidity, and abundant access to food and water. STZ was purchased from Sigma (USA). Antibodies were purchased from Abcam (UK) or Santa Cruz (USA). All other reagents were procured from standard commercial suppliers unless otherwise noted. 

### 2.2. Preparation and Assay of FXT

FXT, a Chinese patent drug provided by Zhongsheng Pharmaceutical Co, Ltd, Guangdong, China, was produced as described in the Chinese Pharmacopoeia 2010. The main peaks in HPLC profile of FXT were identified to be notoginsenoside R_1_, ginsenoside Rg_1_ and Rb_1_, cryptotanshinone, tanshinone I, harpagoside, and astragaloside IV. The content of these constituents in FXT was determined to be 3.27 mg/g, 15.7 mg/g, 15.5 mg/g, 26.6 *μ*g/g, 31.3 *μ*g/g, 69.0 *μ*g/g, and 0.23 mg/g, respectively, by HPLC.

### 2.3. Induction of Diabetes, Animal Grouping, and FXT Treatment

After 16 hours of fasting, rats received a single dose of 60 mg/kg STZ (in 10 mM sodium citrate buffer, pH 4.5) by intraperitoneal injection. Control rats received citrate buffers alone. After one week, STZ treated rats with blood glucose levels greater than 16.7 mmol/L were considered to be diabetic and randomly divided into three groups: (1) STZ group, 70 rats; (2) FXT 1.05 g/kg group, 60 rats; (3) FXT 0.525 g/kg group, 60 rats. And then FXT-diabetic rats received FXT 1.05 g/kg or 0.525 g/kg by intragastric gavage once daily for consecutively 24 weeks. FXT was dissolved in 0.5% CMC and the daily dosage of drug was 1 mL/100 g weight. Control (30 rats) and untreated STZ rats received the isometric 0.5% CMC the same way.

Fasting body weight and fasting blood glucose were recorded every four weeks throughout the study. The general physical signs, including coat color, skin, bite and sup, urine and stool, and crystalline lens, were observed and recorded carefully. Blood samples were collected at the end of experiments for hemorheology test.

### 2.4. Trypsin Digest Method and Morphology Studies

Trypsin digestion of the retina was performed according to the method of Cogan et al. [19] with some modifications. The retina was isolated and incubated at 37°C in digestion buffer (0.1 mol/L Tris buffer, pH 7.8), containing 3% crude trypsin (Difco Laboratories, Detroit, MI, USA), in an air bath oscillator. After 2-3 h of incubation, when the internal limiting membrane began to separate from the retina, the retina was transferred to phosphate-buffered saline (pH 7.4) at room temperature. The vascular tree was washed to be free of remaining neural tissue in distilled water under microscopic observation. The preparations were set onto glass slides, air-dried, and stained with haematoxylin and Periodic Acid-Schiff stain (PAS) to evaluate microvascular lesions.

**Figure 1:**
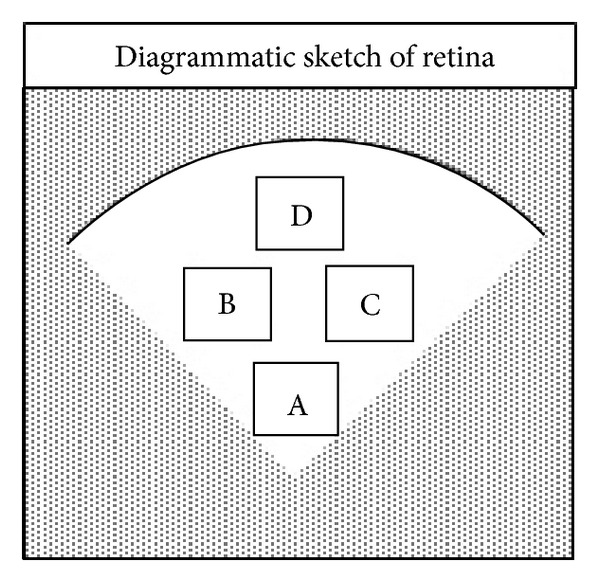


As indicated in Figure 1, we measured the retinal arteriolar caliber and retinal venular caliber in area A of the retina (close to the optic nerve) by using the Image Pro Plus Analysis Software (developed by Media Cybernetics, USA) and then calculated the arteriolar-venular ratio (AVR). The number of pericytes was counted in areas B and D of the retina using an image system (ECLIPSE 80i, Nikon, Japan) and was normalized to the relative capillary density (numbers of cells per millimeter squared of capillary area). To assess the endothelial lesion, the acellular-occluded vessel segments were counted in areas C and D of the retina.

### 2.5. Hemorheological Measurements

Fresh blood samples were collected from rats by arteriopuncture into liquaemin (10 mg/mL) test tubes. Plasma was separated from blood by centrifugation at 3000 r/min for 15 min. Whole blood viscosity and plasma viscosities were measured using a blood viscometer (developed by STEELLEX, China). The serial blood viscosities at different shear rates, 1, 60, and 200 s^−1^, respectively, were determined via a testing program. Erythrocyte aggregability was measured by RBC Deformation and Aggregation Test Instrument. (STEELLEX, China).

### 2.6. Western Blot Analysis

Western blot analysis was performed as described by Park et al. [20] and Chen et al. [21]. The expression of occludin and ICAM-1 was normalized by *β*-actin. Rabbit anti-occludin antibody (ab31721, 1 : 500 dilution, Abcam, HK) and mouse anti-intercellular adhesion molecule (ICAM)-1 antibody (ab2213, 1 : 2000 dilution, Abcam, UK), goat anti-rabbit lgG (1 : 500), and goat anti-mouse lgG (1 : 2000) were used in the study. The proteins were visualized with ECL, kit (Sc-2048, Santa Cruz). The Image J Analysis Software (developed by National Institutes of Health, USA) was used to analyze the gray intensity of target band.

### 2.7. Immunohistochemical Studies

Immunohistochemical analysis was performed as described by Ogata et al. [16] and Juan et al. [22]. Rabbit anti-VEGF (ab46154, 1 : 50 dilution, Abcam, UK) and goat anti-PEDF antibody (sc16956, 1 : 50 dilution, Santa Cruz, USA) were used as the primary antibodies. Primary antibody was omitted for negative controls. Indirect immunoperoxidase staining was performed using immunostaining reagents (Beijing Zhong Shan-Golden Bridge Biological Technology CO, LTD, China). The Image Pro Plus Analysis Software (developed by Media Cybernetics, USA) was used to make image analysis of every immunostaining side of VEGF and PEDF.

### 2.8. Aldose Reductase Assay

Crude aldose reductase (AR) was prepared from rats retinas of each group (*n* = 8). The retina was dissected by a posterior approach and homogenized in 10 volumes of 100 mM potassium phosphate buffer (pH 6.2). The homogenate was centrifuged at 15,000 r/min for 30 min and the supernatant was kept in −20°C refrigerator. The protein concentration of the supernatant was determined using Bradford Protein Assay (Biomiga). AR activity was assayed according to the method described by Akileshwari et al. [23]. The assay mixture was incubated at 37°C and the reaction was initiated by the addition of NADPH at 37°C for 5 min. And then 0.3 mmol/L NaOH 2 mL was added to the mixture at 60°C for 15 min to stop the reaction. The absorbance at 340 nm due to NADPH oxidation was recorded in a spectrophotometer. The activity unit of AR (U) was defined by the absorbance decrease of 0.001 per min of 1 mg protein.

### 2.9. Statistical Analysis

All values were expressed as mean ± SD. The results were analyzed by one-way ANOVA followed by a LSD post hoc test for multiple comparisons. A value of *P* < 0.05 was predetermined as the criterion of significance.

## 3. Results

### 3.1. FXT Shows No Effect on Body Weight Gain and Blood Glucose Level in STZ Rats

**Figure 2:**
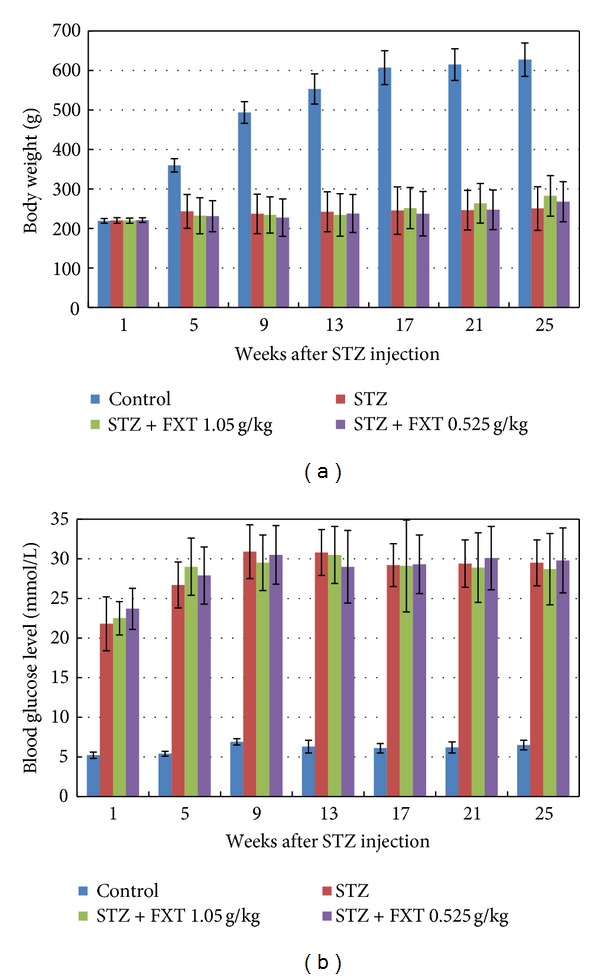
Effects of FXT on body weight and blood glucose level in STZ rats. Diabetic rats received a single dose of 60 mg/kg STZ by i.p. Control rats received vehicle. After one week, STZ rats with blood glucose levels greater than 16.7 mmol/L were assigned into STZ group, FXT 0.525 g/kg group, and FXT 1.05 g/kg group. FXT-diabetic rats received FXT 0.525 g/kg or FXT 1.05 g/kg by ig once daily for consecutively 24 weeks. Fasting body weight (a) and fasting blood glucose level (b) were recorded every four weeks throughout the study. The values are means ± SD. *P < 0.05 versus control rats; ^#^ *P* < 0.05 versus STZ rats.

As shown in Figure 2(a) STZ treatment resulted in a significant decrease of body weight gain during the 24 weeks observation although a slight increase was observed. STZ treatment also induced a sustained high blood glucose level as shown in Figure 2(b). FXT treatment, either 0.525 g/kg or 1.05 g/kg, had no effect on the changes of body weight gain and blood glucose level in rats induced by STZ.

### 3.2. FXT Partially Reduces the Lens Opacities in STZ Rats

To monitor the diabetic cataract, the transparency of the lens was checked every week. Starting from the fourth week, lens opacities appeared in some STZ treated rats. At the end of the trial, we examined the lens of all rats. As shown in Table 1, percentage of STZ rats with both lens opacities was 92.2%, while 80% and 86.1% for FXT 1.05 g/kg and FXT 0.525 g/kg treated rats, respectively.

### 3.3. FXT Extenuates the Microvasculature Lesion Induced by STZ Treatment

**Figure 3:**
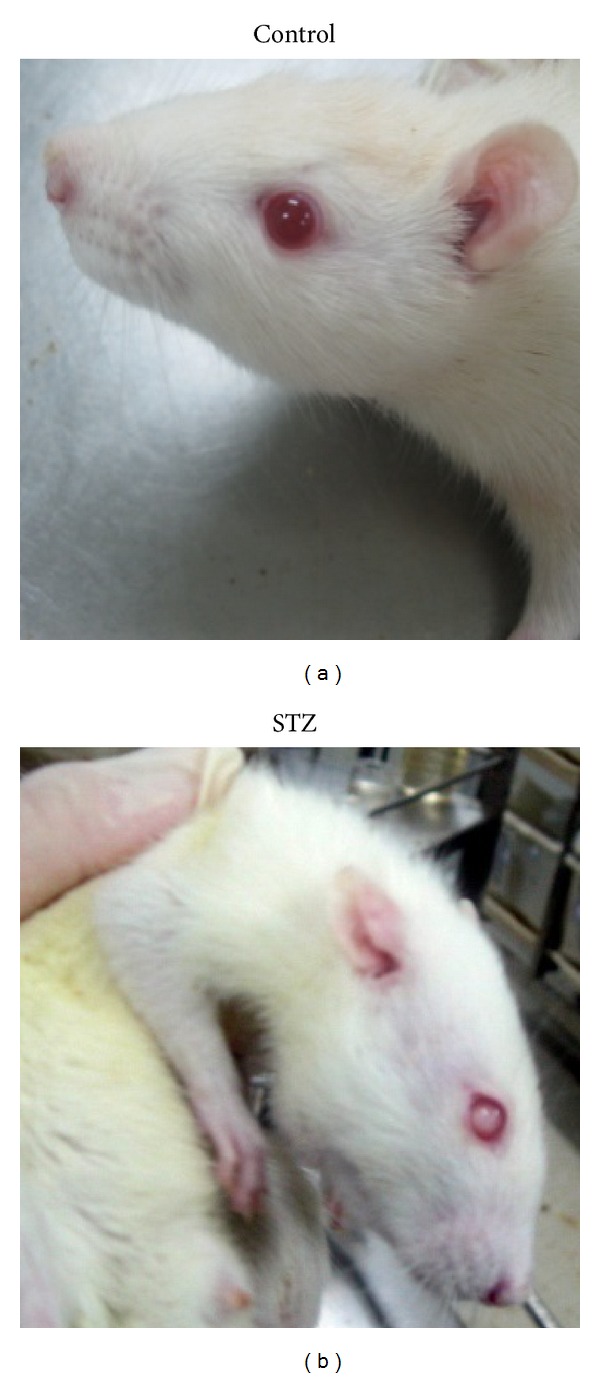
STZ induced lens opacity. Diabetic rats received a single dose of 60 mg/kg STZ by i.p. After one week, STZ rats with blood glucose levels greater than 16.7 mmol/L were assigned into STZ group. Changes of lens opacities were monitered everyweek. Starting from the 4th week of group assignment, the lens opacities appeared in STZ animals. The pictures showed typical clear lens in control rats and opacity lens in STZ rats.

After 24 weeks, the retinal capillaries of STZ rats were strikingly abnormal comparing with those in the control ones. There were abundant acellular vessels with a significant degree of capillary closure in the retina of STZ rats. The retinal capillaries of FXT-treated STZ rats were less affected compared with STZ treated alone, with relatively normal width and cellular integrity as shown in Figure 3. No evidence of capillary microaneurysms was observed in the retina of all animals.

**Figure 4:**
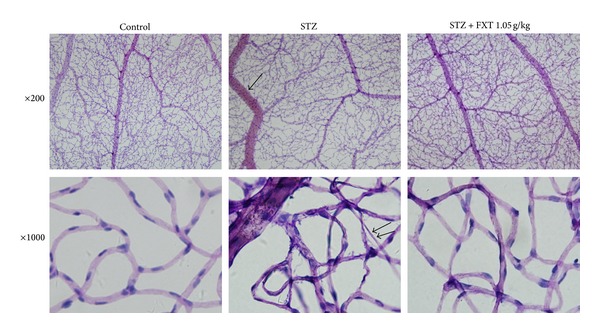
Effects of FXT on retinal microvascular morphology in STZ rats. Diabetic rats received a single dose of 60 mg/kg STZ by i.p. Control rats received vehicle. After one week, STZ rats with blood glucose levels greater than 16.7 mmol/L were assigned into STZ group, FXT 0.525 g/kg group, and FXT 1.05 g/kg group. FXT-diabetic rats received FXT 0.525 g/kg or FXT 1.05 g/kg by ig once daily for consecutively 24 weeks. The retina were prepared with trypsin digestion and stained with haematoxylin and Periodic Acid-Schiff stain. Typical retinal microvascular staining was shown in Figure 4. Vasodilatations in the retinal venula (arrow) and acellular capillaries (double arrow) were observed in STZ rats. Treatment with FXT 1.05 g/kg inhibited the venular dilation and reduced the number of acellular capillaries.

Quantitative analysis demonstrated that the retinal venular caliber in STZ rats significantly increased, with 88.90 ± 2.07 *μ*m versus 75.46 ± 2.65 *μ*m in control rats (*P* < 0.05), indicating significant retinal venular dilatation, which was also supported by arteriolar-venular ratio as listed in Table 2. Normally there was no acellular capillary in retina; however, we observed a remarked increase of acellular vessels in STZ rats, accompanied with obvious pericytes loss as shown in Figure 4 and Table 3. FXT 0.525 g/kg and FXT 1.05 g/kg attenuated the microvasculature lesion with significant reductions of acellular vessels and relative increases of pericytes comparing with STZ treated alone.

### 3.4. FXT Reduces the Blood Viscosity and Erythrocytes Aggregation in STZ Treated Rats

The whole blood viscosity at three different shear rates and the plasma viscosity increased significantly after STZ treatment. The erythrocytes aggregability also elevated in STZ animals. FXT 1.05 g/kg and FXT 0.525 g/kg significantly inhibited the alterations in erythrocytes aggregation and blood viscosity induced by STZ treatment as shown in Table 4.

### 3.5. FXT Reduces ICAM-1 and Increases Occludin in Retina of STZ Treated Rats

**Figure 5:**
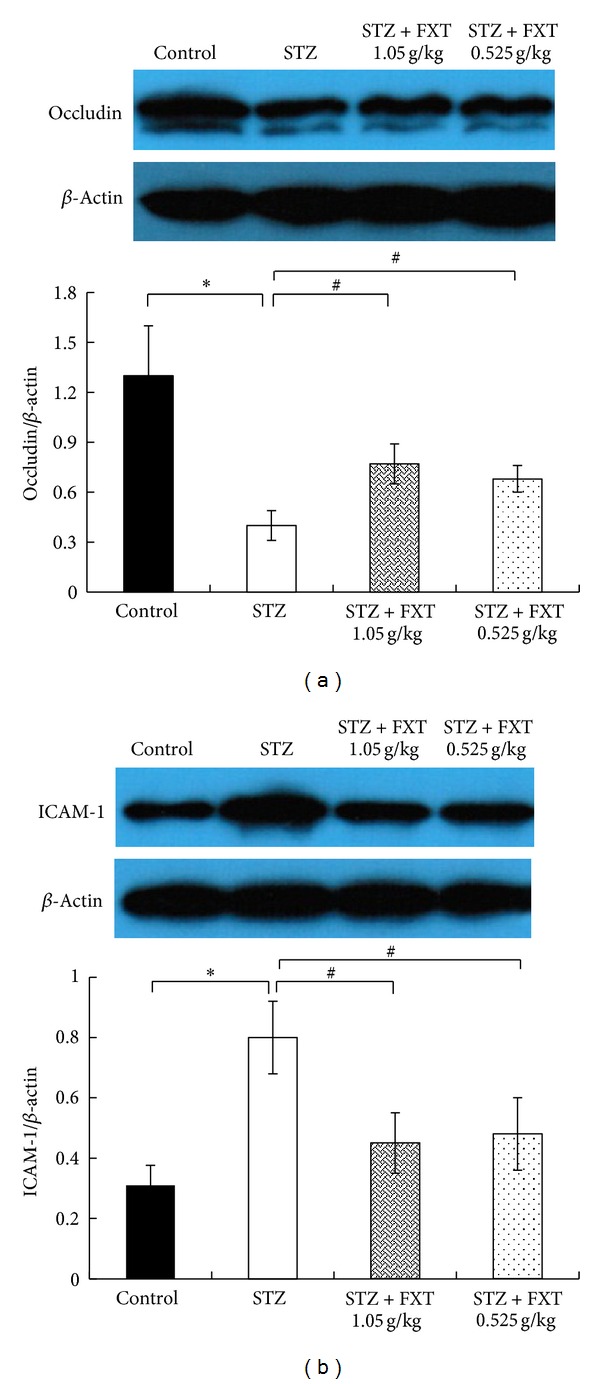
Effects of FXT on the expression of occludin and ICAM-1 of retina in STZ rats. Diabetic rats received a single dose of 60 mg/kkg STZ by i.p. Control rats received vehicle. After one week, STZ rats with blood glucose levels greater than 16.7 mmol/L were assigned into STZ group, FXT 0.525 g/kg group, and FXT 1.05 g/kg group. FXT-diabetic rats received FXT 0.525 g/kg or FXT 1.05 g/kg by ig once daily for consecutively 24 weeks. Occludin and ICAM-1 in retina were detected with western blot. The expression of occludin (a) and ICAM-1 (b) was quantified using densitometry and normalized by *β*-actin. Values are demonstrated as mean ± SD; *n* = 8; *P < 0.05 versus control rats; ^#^ *P* < 0.05 versus STZ rats.

Western blot analysis demonstrated that the retina ICAM-1 significantly increased and occludin decreased after STZ treatment, indicating an obvious inflammation in retina and a possible blood-retinal barrier lesion as shown in Figure 5. Treatment with FXT 0.525 g/kg and 1.05 g/kg not only reduced the ICAM-1 expression but also enhanced the occludin in the retina.

### 3.6. FXT Attenuates the Expression of VEGF and PDEF in Retina Induced by STZ Treatment

**Figure 6:**
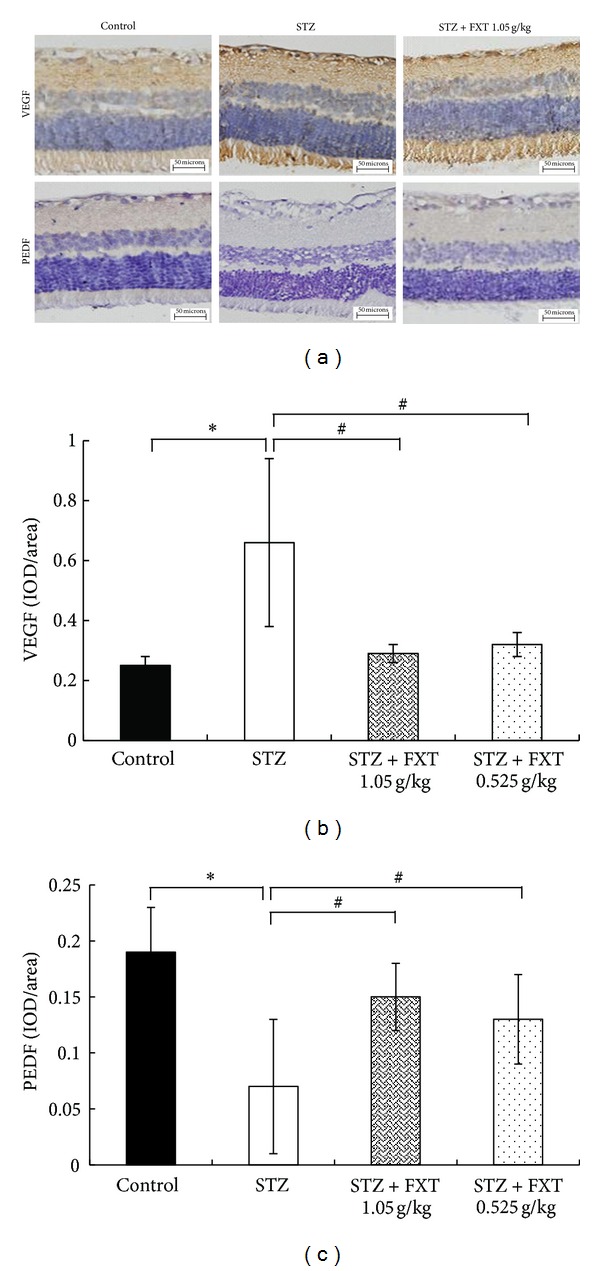
Effects of FXT on the expression of VEGF and PEDF of retina in STZ rats. Diabetic rats received a single dose of 60 mg/kg STZ by i.p. Control rats received vehicle. After one week, STZ rats with blood glucose levels greater than 16.7 mmol/L were assigned into STZ group, FXT 0.525 g/kg group, and FXT 1.05 g/kg group. FXT-diabetic rats received FXT 0.525 g/kg or FXT 1.05 g/kg by ig once daily for consecutively 24 weeks. Typical immunohistochemistry staining for VEGF and PEDF in retina was shown in panel (a). The optical density of VEGF and PEDF was quantified using the Image Pro Plus Analysis Software as shown in panels (b) and (c). *n* = 5. *P < 0.05 versus control rats; ^#^ *P* < 0.05 versus STZ rats.

Immunohistochemistry studies have shown that in control rats VEGF was localized to the pigment epithelium layer (PEL), the outer nuclear layer (ONL), the outer plexiform layer (OPL), inner plexiform layer (IPL), the ganglion cell layer (GCL), and also the nerve fiber layer (NFL), while PEDE was mainly localized to the PEL and the inlayers of retina. STZ treatment caused a strong upregulation of VEGF in all layers of retina, particularly in PEL, the inner nuclear layer (INL), and GCL; however, STZ induced an overall decrease in PEDE expression. As shown in Figure 6, FXT 0.525 g/kg and FXT 1.05 g/kg treatment significantly attenuated the alterations in VEGF and PEDE expression induced by STZ though not the same as that in control rats.

### 3.7. FXT Decreases the Activity of Retina Aldose Reductase in STZ Treated Rats

**Figure 7:**
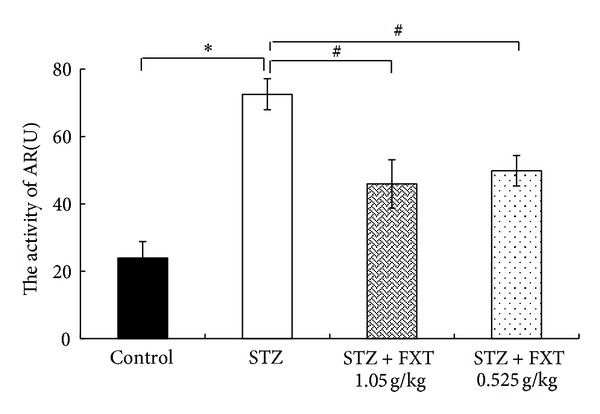
Effects of FXT on the activity of aldose reductase of retina in STZ rats. Diabetic rats received a single dose of 60 mg/kg STZ by i.p. Control rats received vehicle. After one week, STZ rats with blood glucose levels greater than 16.7 mmol/L were assigned into STZ group, FXT 0.525 g/kg group, and FXT 1.05 g/kg group. FXT-diabetic rats received FXT 0.525 g/kg or FXT 1.05 g/kg by ig once daily for consecutively 24 weeks. The activity of aldose reductase of retina was detected with NADPH oxidation method. The values are means ± SD. *n* = 8.  *P < 0.05 versus control rats; ^#^ *P* < 0.05 versus STZ rats.

Increased aldose reductase activity is one of the important initiating factors in diabetic retinopathy. As shown in Figure 7, there was a significant increase of aldose reductase activity in the retina 24 weeks after STZ treatment (*P < 0.05 versus control). FXT 0.525 g/kg and FXT 1.05 g/kg effectively attenuated the enzyme activity increase induced by STZ though still higher than that in controls rats.

## 4. Discussion

The aim of the study was to investigate the effects of FXT on microvessel lesion on early stage of diabetic retinopathy. The results suggest that FXT 0.525 g/kg and 1.05 g/kg have distinct protective effect on STZ induced microvessel injury.

When the blood glucose persistently increases, the activity of aldose reductase in pericytes of retinal capillaries increases too, resulting in enhanced level of metabolites (sorbitol and fructose) in intracellular pericytes leading to an elevation of intracellular osmotic pressure,cell swelling and metabolic disorders, eventually bringing about the loss of pericytes and damage of their primary function (autoregulation of retinal capillaries) [11, 12]. Without the protection of pericytes, the proliferation of endothelial cells will result in saccular outpouching of capillary walls, leading to microaneurysms with hemorrhage tendency [24]. We found that diabetic rats that received FXT-treatment had decreased activity of aldose reductase in retina. At the same time, the number of retina pericytes in FXT treated rats increased comparing with those of STZ alone, indicating a possible mechanism that the protection of FXT on pericytes may attribute to its action of reducing aldose reductase activity. Certainly, mechanisms concerning the loss of pericytes in retinal capillaries are complicated and stimulating of aldose reductase pathway may not be the only ones.

Elevated glycosylated hemoglobin in red blood cell (RBC) triggered by increased blood glucose results in cellular stiffening, decreasing their deformation capacity which leads to an increase in blood viscosity and a constant, elevated sheer stress at the endothelium of retinal vessel. The integrity of blood vessel could be damaged by increased sheer stress together with pericytes loss [25, 26]. The subsequent hypoxia results in compensatory expansion of retinal vessels to increase perfusion [27]. We found that FXT treatment significantly restrained the retinal veins dilation and protected the microvessel architecture lesion induced by STZ. The protective effect of FXT at least partially attributes to its effect on blood hemorheology because we observed a significant decline in whole blood viscosity, plasma viscosity, and erythrocyte aggregation after FXT treatment.

VEGF plays a key role in the onset and development of diabetic retinopathy [28, 29]. Accumulating pieces of evidence suggest that overexpression of VEGF could damage the structure of BRB by stimulating the increase of ICAM-1, which leads to leukocyte adherence to endothelial cells and triggers inflammatory lesion [30, 31]. We found that VEGF was overexpressed in retina after STZ insult, which covered nearly every layer of retina, accompanyied with elevated expression of ICAM-1 and decreased occludin in retina, which is a key component of BRB structure [32, 33]. The results suggested an obvious inflammation and BRB lesion in diabetic rats. The protective effect of FXT on BRB integrity might be related to its effect of inhibiting VEGF and ICAM-1 expression.

Angiogenesis in retina is a hallmark of diabetic retinopathy turning into proliferative phase, which can be controlled by inhibitors. Admittedly, VEGF is the most important mediator in triggering angiogenesis and there are large numbers of endogenic angiogenic inhibitors. As the most effective and natural angiogenic inhibitor, PEDF has better control effect on angiogenesis than angiotensin, thrombospondin-1, and other angiogenic inhibitors [16]. The balance between VEGF and PEDF determines the proliferation of angiogenesis in diabetic retinopathy. In our study, decreased expression of PEDF in retina in diabetic rats suggested that the balance between VEGF and PEDF was disrupted. In our study, PEDF was elevated in retina of diabetic rats that received FXT 1.05 g/kg and FXT 0.525 g/kg treatment, respectively, 2.1 times and 1.9 times. The results indicated that the improvement of FXT on diabetic retinopathy may be connected with its effects on the balance of VEGF and PEDF.

Diabetic cataract is another diabetic eye diseases, whose harm ranks only second to diabetic retinopathy [34–36]. Diabetic cataract is characterized by lens edema, protein synthesis, destroyed cell structures, and lens opaque. In the study, we found that starting from the fourth week, lens opacities appeared in some STZ rats and FXT rats. At the end of the trial, percentage of FXT 1.05 g/kg and FXT 0.525 g/kg treated rats was inferior to STZ rats. It indicated that FXT 1.05 g/kg and FXT 0.525 g/kg may decrease the lens opaque rate in diabetic rats and the mechanism needs further investigation.

Diabetic retinopathy, a microcirculation disturbance triggered by a persistent increase in blood glucose levels, is a multifactorial, complicated illness expressed in abnormality of vessel and blood in retina. In this study we found that FXT has protective effect on STZ induced diabetic retinopathy in rats with possible mechanisms of inhibiting AR activity and VEGF expression. However, there are lots of questions need to be answered before we elucidate the mechanisms of FXT protecting the diabetic retina. 

## Figures and Tables

**Table 1:** Effects of FXT on lens opacities in STZ diabetic rats.

Group	Animals (*N*)	Both lens clear *N* (%)	Both lens opacities *N* (%)
Control	30	30 (100)	0 (0)
STZ	51	3 (5.9)	47 (92.2)
STZ + FXT 1.05 g/kg	40	6 (15)	32 (80)
STZ + FXT 0.525 g/kg	36	2 (5.6)	31 (86.1)

**Table 2:** Effects of FXT on retinal vessel caliber in STZ diabetic rats ($\overset{-}{x}\pm \text{SD}$).

Group	Rats (*N*)	Retinal arteriole caliber (*μ*m)	Retinal venular caliber (*μ*m)	Arteriolar-venular ratio (AVR)
Control	9	43.83 ± 0.56	75.46 ± 2.65	0.58 ± 0.02
STZ	18	42.45 ± 1.04	88.90 ± 2.07*	0.48 ± 0.08*
STZ + FXT 1.05 g/kg	9	42.11 ± 1.65	79.92 ± 2.87^#^	0.53 ± 0.05^#^
STZ + FXT 0.525 g/kg	10	43.06 ± 0.96	80.24 ± 2.44^#^	0.54 ± 0.07^#^

The values are means ± SD. *P < 0.05 versus control rats; ^#^ *P* < 0.05 versus STZ rats.

**Table 3:** Effects of FXT on the number of retinal pericyte and acellular capillaries in STZ diabetic rats ($\overset{-}{x}\pm \text{SD}$).

Group	Rats (*N*)	Pericyte numbers/mm^2^	The number of acellular capillaries/mm^2^
Control	18	762.2 ± 62.2	0
STZ	36	572.2 ± 117.4*	15.5 ± 6.45
STZ + FXT 1.05 g/kg	18	654.4 ± 140.8	10.2 ± 2.82^#^
STZ + FXT 0.525 g/kg	20	607.8 ± 97.9	11.4 ± 3.71^#^

The values are means ± SD. *P < 0.05 versus control rats; ^#^ *P* < 0.05 versus diabetic rats.

**Table 4:** Effects of FXT on erythrocyte aggregation, blood and plasma viscosity in STZ diabetic rats ($\overset{-}{x}\pm \text{SD}$, *n* = 12).

Group	Erythrocyte aggregation index	Whole blood viscosity (mPa/s)			Plasma viscosity (mPa/s)
		*γ* = 200 (1/s)	*γ* = 60 (1/s)	*γ* = 1 (1/s)	*γ* = 60 (1/s)
Control	0.85 ± 0.22	4.05 ± 0.34	4.64 ± 0.28	23.54 ± 2.23	0.97 ± 0.04
STZ	1.44 ± 0.69*	4.41 ± 0.32*	5.34 ± 0.37*	27.74 ± 2.63*	1.09 ± 0.18*
STZ + FXT 1.05 g/kg	1.03 ± 0.19^#^	4.31 ± 0.38	4.96 ± 0.37^#^	25.36 ± 3.20^#^	1.03 ± 0.06
STZ + FXT 0.525 g/kg	1.08 ± 0.25^#^	4.24 ± 0.34	4.88 ± 0.30^#^	25.28 ± 2.79^#^	1.02 ± 0.08

The values are mean ± SD. *P < 0.05 versus control rats; ^#^ *P* < 0.05 versus diabetic rats.
